# Saponin content in medicinal plants in response to application of organic and inorganic fertilizers: a meta-analysis

**DOI:** 10.3389/fpls.2025.1535170

**Published:** 2025-02-11

**Authors:** Junyan Lv, Shouzan Liu, Chunli Hu, Lan Ding, Hongzhen Wang, Xin Li, Feng Yang, Qiong Shen, Hongbin Zhang, Guobiao Ma, Shaobo Zhang, Yan Bai

**Affiliations:** ^1^ College of Food and Health, Zhejiang Agriculture and Forestry University, Hangzhou, China; ^2^ Tea Research Institute, Chinese Academy of Agricultural Sciences, Hangzhou, China; ^3^ State Key Laboratory of Subtropical Silviculture, Zhejiang Agriculture and Forestry University, Hangzhou, China; ^4^ Food and Drug Inspection and Testing Center, Hangzhou, China; ^5^ Agriculture and Forestry Technology Extension Center, Hangzhou, China

**Keywords:** fertilizers, meta-analysis, medicinal plant, saponins, bioactive compounds

## Abstract

The application of fertilizers is a key agronomic practice in the artificial cultivation of medicinal plants, aiming to boost yields and enhance the levels of their bioactive constituents. However, systematic investigations on the influence of various fertilizers on the concentration of active compounds in saponin-containing medicinal plants remain insufficient. In this study, 966 experimental outcomes from 29 papers were analyzed via meta-analysis to examine the effects of organic fertilizers, inorganic fertilizers, and their combined application on the levels of different saponin monomers in medicinal plants. The findings from the meta-analysis revealed that inorganic fertilizers contribute positively to the accumulation of saponins such as Rg1 in ginseng, Rb1, Rc, Rd, Re, and Rg1, in addition to the saponins from *Paris polyphylla*, *Dioscorea*, *Panax quinquefolius*, and *Platycodon grandiflorus*. Moreover, the application of organic fertilizers was found to markedly elevate the concentrations of Notoginsenoside R1, Ginsenoside Rb1, Ginsenoside Rb2, Re, and Rg1, along with Lancemasid saponins and Quinoa saponins. The combined use of both organic and inorganic fertilizers was shown to effectively increase the levels of Notoginsenoside R1 and *Panax ginsenosides*, encompassing Rb1, Rb2, Rc, Rd, Re, and Rg1. Overall, the results suggest that both individual and combined applications of organic and inorganic fertilizers have a positive impact on the enhancement of saponin monomers in medicinal plants. However, inorganic fertilizers promote the increase of saponin content, their prolonged use may lead to soil compaction and acidification, which could compromise the yield and quality of medicinal plants. On the other hand, organic fertilizers improve the soil environment and stimulate saponin accumulation, they do not supply all the nutrients required for the sustained growth of these plants. Therefore, a balanced fertilization strategy combining both organic and inorganic fertilizers is recommended as the optimal approach for cultivating saponin-rich medicinal plants.

## 1 Introduction

Traditional Chinese Medicine (TCM) holds a significant position in the Chinese healthcare system, serving a critical function in the treatment of a wide range of ailments, including both internal and external disorders, as well as insect and snake envenomations (Bao et al., 2022; Ai et al., 2023; Liu et al., 2023a). With the increasing recognition of the therapeutic benefits of TCM, the demand for Chinese medicinal materials has been steadily rising (Atherton and Li, 2023; Yin et al., 2024). However, the slow growth rate of wild medicinal plants, coupled with excessive harvesting, has created substantial challenges in meeting market demands. As a result, the expansion of artificial cultivation has become a key strategy to alleviate this issue (Tian et al., 2023; Zhang et al., 2023). Although artificial cultivation offers a potential solution to the scarcity of wild resources (Chen et al., 2024a), practices such as over-fertilization and improper fertilizer selection may adversely affect the quality and potency of bioactive compounds in medicinal plants while also degrading soil health, thus hindering the sustainable development of cultivated TCM plants (Cun et al., 2023; Li et al., 2023e). Therefore, it is essential to conduct a thorough investigation into the impact of various fertilizers on the quality of TCM in order to promote the scientific cultivation of these plants.

The application of fertilizers is a commonly employed agronomic practice aimed at enhancing both the yield and quality of Chinese medicinal plants (Chung et al., 2010; Gavrić et al., 2021; Loera-Muro et al., 2021; Wei et al., 2024). Fertilizer application can rapidly increase nutrient availability in the soil, fulfilling the short-term demands for nitrogen, phosphorus, and potassium during the growth and development of medicinal plants, thus promoting improved yields and overall plant quality (Zhang et al., 2020; Liu et al., 2022; Liu et al., 2024a). Inorganic fertilizers, characterized by their pure nutrient composition, high nutrient concentration, and rapid efficacy, supply essential nutrients for plant growth, development, and the synthesis of secondary metabolites within a relatively brief timeframe (Zhao et al., 2021; Sha et al., 2022; Liu et al., 2024a; Wan et al., 2024). Previous studies have demonstrated that the application of nitrogen fertilizers markedly boosts the accumulation of bioactive compounds in root and rhizome-based medicinal plants (Clemensen et al., 2017). For instance, Wei et al. (2020) reported that the use of inorganic nitrogen fertilizers substantially enhances the growth of Sanqi roots while increasing the concentration of secondary metabolites. Similarly, Economakis et al. (2002) found that the addition of phosphorus fertilizer effectively promotes the accumulation of volatile compounds in oregano, while Lee et al. (2005) observed that phosphorus fertilizer application markedly increases the concentration of sesquiterpenes in chrysanthemum. Nevertheless, the unregulated use of inorganic fertilizers may adversely affect plant growth and development by altering soil structure, pH, and other factors, thereby compromising both yield and quality (Appelhans et al., 2020; Geetha et al., 2023). Excessive nitrogen application, for example, can lead to soil acidification and plant lodging, which in turn may reduce the content of bioactive components (Sainju et al., 2019; Gu et al., 2021; Wang et al., 2022; Zhang et al., 2022, Zhang et al., 2024). Although potassium fertilizers enhance a plant’s ability to absorb water and nutrients, excessive use can result in a decrease in medicinal plant quality (Yang et al., 2021). In summary, inorganic fertilizers can provide the necessary nutrients for rapid medicinal plant growth, leading to increased yield and improved quality; however, their prolonged and improper use may have detrimental effects on the sustainable development of cultivated medicinal plants.

Compared to inorganic fertilizers, organic fertilizers are composed of a broader array of nutrients that address the diverse nutritional needs of plants (Dai et al., 2021; Li et al., 2023c; Liu et al., 2024a). These fertilizers can enhance soil structure and nutrient content while stimulating microbial activity, thereby promoting the growth, development, and synthesis of secondary metabolites in medicinal plants (Shi et al., 2022; Budiastuti et al., 2023; Hou et al., 2023; Yu et al., 2024). For instance, Liu et al. (2024b) demonstrated that the combined application of biochar and organic fertilizer substantially increased both biomass and the levels of key secondary metabolites (matrine and oxymatrine) in Sophora by regulating soil microorganisms. Another study by Dong et al. (2019) revealed that incorporating pig and cow manure in a 1:2 ratio resulted in a 17.0–19.1% increase in ginsenoside content. However, previous research has shown that organic fertilizers typically exhibit lower nutrient concentrations and slower nutrient release rates, requiring an extended period for crop absorption and utilization, which poses challenges in meeting the high-yield demands of crops within a short time frame (Chatzistathis et al., 2021; El Moussaoui et al., 2023). In contrast, the combined application of organic and inorganic fertilizers not only meets the immediate nutrient needs for medicinal plant growth but also sustains their long-term nutritional requirements (Yang et al., 2020; He et al., 2024). Organic fertilizers can also alleviate issues such as soil acidification and compaction caused by inorganic fertilizers, thereby improving soil health and supporting medicinal plant growth (Niu et al., 2022; Wang et al., 2024b; Xing et al., 2024). Wei et al. (2024) found that substituting chemical fertilizers with organic fertilizers during *Pinellia* cultivation led to increased yields and enhanced growth parameters. In conclusion, the application of fertilizers is a key factor in boosting the yield and quality of medicinal plants. Different fertilizer types exert varying effects on these outcomes, highlighting the necessity for comprehensive research into the impacts of sole organic and inorganic fertilizer applications, as well as their combined use, on the quality of medicinal plants to promote the scientific cultivation of Chinese medicinal plants.

Saponin-containing medicinal plants hold a crucial position in TCM, demonstrating a broad spectrum of pharmacological effects, such as anti-inflammatory, immune-modulating, anticancer, antiviral, antioxidant, anti-fatigue, and cardiovascular enhancement properties. Prominent examples of saponin-containing medicinal plants include *Panax notoginseng*, *Panax ginseng*, *Bupleurum chinense*, and *Rehmannia glutinosa* (Yang et al., 2019; Hawthorne et al., 2022; Zhou et al., 2024). Due to the depletion of wild resources, artificial cultivation has emerged as the predominant approach to satisfy market demand demands (Atherton and Li, 2023; Yin et al., 2024). Fertilizer application, as an important agronomic practice, has been shown to markedly improve the yield and quality of saponin-rich medicinal plants (Moon et al., 2018; Saha et al., 2019; Bao et al., 2022). For examples, the research showed that the application of 45.48-53.83 kg hm^-^² nitrogen, 179.98-236.83 kg hm^-^² phosphorus and 29.80-39.95 kg hm^-^² potassium in the field soil could significantly increase the saponin content in the *Paris polyphylla* var. *chinensis* by 11.09% (Liu et al., 2019). And the results of Moon et al. (2018) found that the application of organic fertilizers such as mixed organic matter and fermentation cake can significantly increase the content of the main saponin, lancemaside A, in *Codonopsis lanceolata.* However, inappropriate cultivation practices, especially unscientific fertilizer use, can exacerbate cropping obstacles and diseases, which can negatively affect the quality and yield of medicinal plants (Hu et al., 2016; Chen et al., 2022; Geetha et al., 2023; Thakur et al., 2023). Therefore, it is very important to choose scientific fertilization methods to improve the yield and quality of medicinal plants and promote their sustainable development. In sum, this study systematically evaluates 966 experimental results drawn from 29 published studies, specifically focusing on the impact of organic fertilizers, inorganic fertilizers, and their combined application on the content of saponin monomers in medicinal plants. The primary objective is to consolidate these findings and formulate general conclusions concerning the effects of fertilizer additions—both organic and inorganic—on saponins in medicinal plants. The specific aim is to provide scientific evidence and guidance for the optimal selection and application of fertilizers in the cultivation of saponin-containing medicinal plants.

## 2 Materials and methods

### 2.1 Research literature retrieval

A systematic search was conducted across multiple databases to identify studies that met the predefined inclusion criteria. The Web of Science database was utilized for locating literature in English, while the Wan Fang Data and China National Knowledge Infrastructure databases were employed to retrieve Chinese-language literature. The search strategy incorporated both controlled vocabulary and free-text terms in each database. The following subject terms were included in the search: “Saponins” [Mesh], “Ginsenosides” [Mesh], “Escin” [Mesh], “Quillaja Saponins” [Mesh], “Dioscin” [Mesh], “Polyphyllin” [Mesh], and “Saikosaponins” [Mesh]. Additional terms such as “Notoginsenoside” [Mesh], “Sarsasapogenin” [Mesh], “Fertilizers” [Mesh], “Nitrogen fertilizer addition” [Mesh], “Phosphorus fertilizer addition” [Mesh], “Potassium fertilizer addition” [Mesh], and “NPK fertilizer addition” were also included, resulting in the retrieval of a total of 1,273 relevant articles.

### 2.2 Characteristics of research literature and data extraction standards

**Figure 1:**
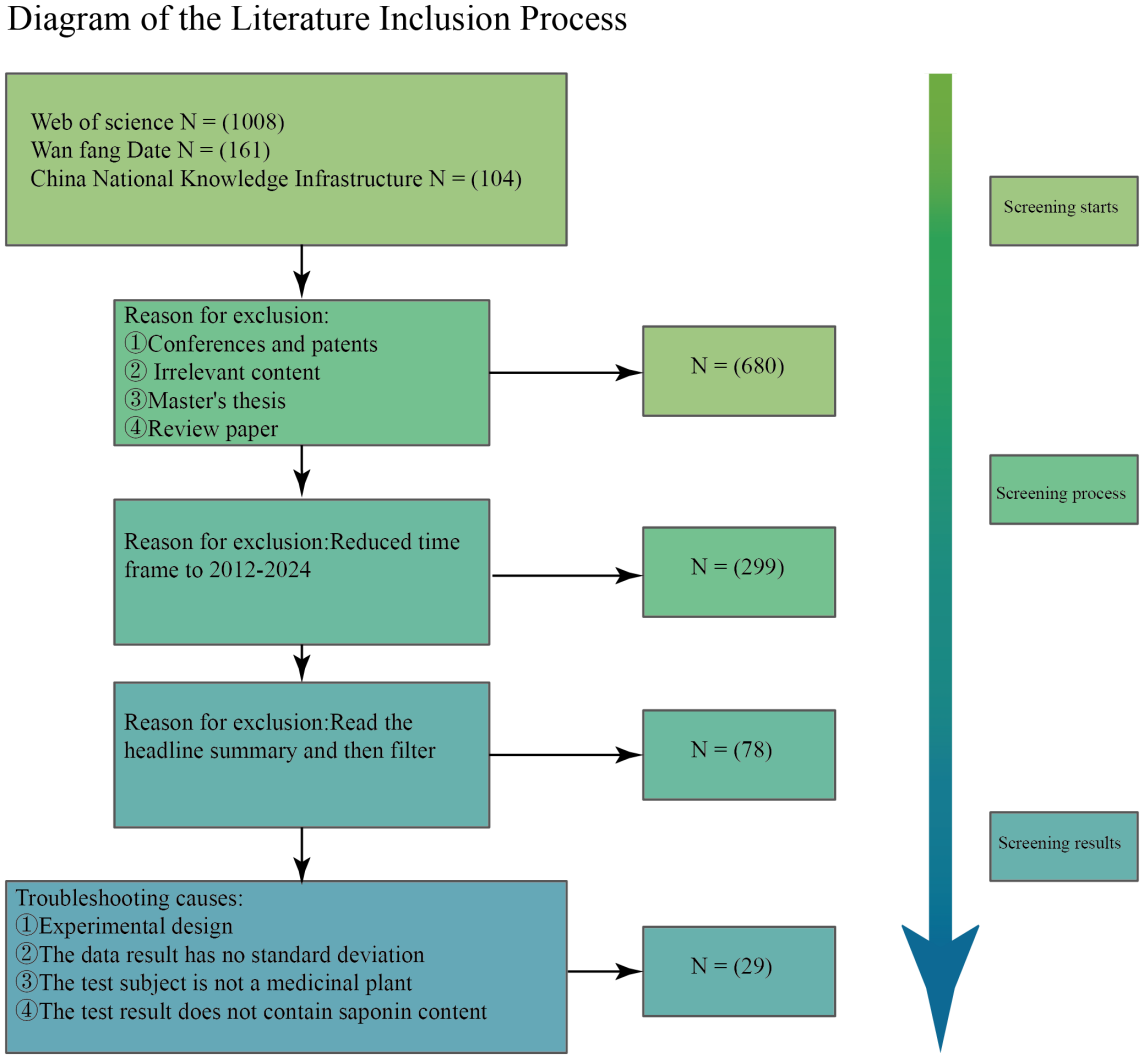
Literature incorporation flow chart, literature data collection and screening process.

The study adhered to a structured series of screening steps, as depicted in **Figure 1**. Initially, a comprehensive search combining both subject-specific and free terms was performed across three databases, yielding a total of 1,273 articles. After eliminating patents, conference papers, and dissertations, 680 articles were retained for further assessment. The search scope was then limited to the years 2012-2024, resulting in 299 articles. Following an in-depth review of the abstracts, 78 articles were identified as relevant to the study. In the final step, 29 articles were selected according to the inclusion criteria, which encompassed a total of 912 research findings. The established criteria for article inclusion were as follows: 1) the experimental subjects were required to be medicinal plants; 2) the experimental group had to utilize either organic or inorganic fertilizers; 3) the articles had to provide detailed information regarding fertilizer application rates and methods; and 4) the experimental results must include saponin content data. Some data were extracted using WebPlotDigitizer-4.7. **Tables 1A**, **1B** provides a summary of the author information for the 29 references, as well as details of the test sites, test subjects (medicinal plants) and specific test methods.

**Table 1A:** Characterization of the effect of nitrogen, N、P、K fertilizer addition on the saponin content of different medicinal plants.

	Authour	Test Object	Test type	Type of climate in the test site	Test location	Type of organic fertilizer added to the experiment	Type of inorganic fertilizer used in the experiment	Organic Fertilizer Base Fertilizer	Inorganic base fertilizer	Whether to apply additional fertilizer	Additional application of organic fertilizer	Additional application of inorganic fertilizers	Saponin types
1	Li et al. (2022)	Panax notoginseng	Field experiment	Subtropical Monsoon	Kunming, Yunnan Province, China	NPK:Decomposed organic fertilizer of plant origin; Carbopeptidin organic fertilizer	—	30000kg/ha 15000kg/ha 7500 kg/ha 15000kg/ha 7500 kg/ha 3750 kg/ha 3000 kg/ha 150 kg/ha 700 kg/ha	—	TRUE	0 kg/ha 15000 kg/ha 7500 kg/ha 3750 kg/ha 1200 kg/ha 600 kg/ha 300 kg/ha	—	Ginsengsaponin Rg1 Ginsengsaponin Rb1 Notoginsenoside R1
2	Ou et al. (2014)	Panax notoginseng	Potting experiment	Humid subtropical seasonal -wind climate	Yunnan Wenshan Panax pseudoginseng Research Institute Yanshan Experimental Farm	—	N:Ammonium nitrate P:Calcium-magnesium phosphate fertilizer K:potassium sulfate Anong Trace Element Nutrient Solution	—	P and K were added at 0.15 ~ 0.30 g/kg soil; Anong trace elements were added at 9 mL/pot; N: 100%, 70%, 50%, 30%, 0%	TRUE	—	N:0%、30%、50%、70%、100%	Ginsengsaponin Rg1 Ginsengsaponin Rb1 Ginsengsaponin Rd1 Notoginsenoside R1
3	Du et al. (2022)	Panax notoginseng	Field experiment	Subtropical Plateau Mountain Monsoon Climate	Haiwei Village, Jinliao Town, Xundian County, Kunming, Yunnan Province, China	—	N:Ammonium nitrate P:Calcium-magnesium phosphate fertilizer K:potassium sulfate、dicalcium chloride	—	P:280 kg/ha N:300 kg/ha K:337.5kg/ha 675kg/ha 1012.5 kg/ha	TRUE	—	—	Ginsengsaponin Rg1 Ginsengsaponin Rb1 Ginsengsaponin Rd Ginsengsaponin Re Notoginsenoside R1
4	Wei et al. (2020)	Panax notoginseng	Field experiment	Subtropical Plateau Mountain Monsoon Climate	Yunnan Agricultural University, Xundian County, Yunnan Province	—	N:UREA P:calcium superphosphate (Ca(OH)_2_) K:potassium sulfate	—	P:(180 kg/ha) K: (270 kg/ha) N:56、113、225kg/ha	TRUE	—	20% of the total amount of each fertilizer	Notoginsenoside
5	Ou et al. (2017)	Panax notoginseng	Potting experiment	Subtropical Monsoon	Wenshan, Yunnan Province, China	Rapeseed after oil extraction	P:Calcium-magnesium phosphate fertilizer K:potassium sulfate N:Trace element nutrient solution	Total organic nitrogen resources, P_2_O_5_, K_2_O and trace elements were applied as base fertilizer at one time	40% Base Fertilizer of Total Inorganic Nitrogen Resources	TRUE	无	60% equivalent topdressing	Ginsengsaponin Rg1 Ginsengsaponin Rb1 Ginsengsaponin Rd Notoginsenoside R1
6	Li et al. (2021a)	Panax notoginseng	Field experiment	Subtropical Plateau Mountain Monsoon Climate	Kunming, Yunnan Province, China	NPK:Organic Fertilizer for Fruits, Vegetables and Chinese Medicine	—	33000kg/ha 45000kg/ha 63000kg/ha	—	TRUE	18000kg/ha15000kg/ha 25500kg/ha25500kg/ha 33000kg/ha30000kg/ha	—	Ginsengsaponin Rg1 Ginsengsaponin Rb1 Ginsengsaponin Rd Ginsengsaponin Re Notoginsenoside R1
7	Zhu et al. (2023)	Panax notoginseng	Potting experiment	Subtropical Plateau Mountain Monsoon Climate	Kunming, Yunnan Province, China	—	N:Compound fertilizer P:calcium superphosphate (Ca(OH)_2_) K:potassium sulfate	P:225kg/ha K:450kg/ha N:450kg/ha 225kg/ha	—	TRUE	P:225kg/ha K:450kg/ha N:450kg/ha 225kg/ha	—	Ginsengsaponin Rg1 Ginsengsaponin Rb1 Ginsengsaponin Rd Ginsengsaponin Re Notoginsenoside R1
8	Wu et al. (2022)	Ginseng	Potting experiment	East Asian monsoon climate zone	Jingyu County Experimental Base, Baishan City, Jilin Province, China	—	K:potassium sulfate N、P:Modified Hoagland nutrient solution	—	50%potassium sulfate(0,2,4,8,10,12 mmol·L-1)	TRUE	—	50% potassium sulfate left(0,2,4,8,10,12 mmol·L-1)	Ginsengsaponin Ginsengsaponin Rg1 Ginsengsaponin Rb1 Ginsengsaponin Re
9	Dong et al. (2019)	Ginseng	Understory biomimetic cultivation experiment	East Asian monsoon climate zone	Jingyu City, Jilin Province, China	NPK:Pig manure: cow manure 1:2	Liquid gel suspensions (Burkholderia and Rhizobia)	30,000 kg/ha	3g/kg 6g/kg 9g/kg 1.5g/kg 3g/kg 4.5g/kg	FALSE	—	—	Ginsengsaponin Rg1 Ginsengsaponin Rb1 Ginsengsaponin Rb2 Ginsengsaponin Rc Ginsengsaponin Rd Ginsengsaponin Re
10	Jang et al. (2020)	Ginseng	Field experiment	Warm temperate maritime climate	Pusan, Korea	—	Na_2_SiO_3_·9H_2_O(Foliar fertilizer)	—	0.05mg/L 0.1mg/L 0.2mg/L	FALSE	—	—	Ginsengsaponin Ap Ginsengsaponin Rb1 Ginsengsaponin Rb2 Ginsengsaponin Rc Ginsengsaponin Re Ginsengsaponin Rg1
11	Yang et al. (2019)	Ginseng	Field experiment	Subtropical Plateau Mountain Monsoon Climate	Kunming, Yunnan Province, China	—	biomass charcoal	—	0g/kg 4g/kg 8g/kg 12g/kg	FALSE	—	—	Ginsengsaponin R1 Ginsengsaponin Rb1 Ginsengsaponin Rd Ginsengsaponin Re Ginsengsaponin Rg1 Ginsengsaponin Rh
12	Tuo et al. (2023)	Ginseng	Field experiment	Subtropical Monsoon	Luxi County, Yunnan Province, China	NPK:Dermet Water soluble fertilizer	—	360kg/ha 288kg/ha 216kg/ha 144kg/ha 432kg/ha 576kg/ha 360kg/ha 288kg/ha	—	FALSE	—	—	Ginsengsaponin R1 Ginsengsaponin Rb1 Ginsengsaponin Rd Ginsengsaponin Re Ginsengsaponin Rg1
13	Kołodziej et al. (2015)	Ginseng	Field experiment	Temperate continental monsoon climate	Lublin, Poland	—	KN	—	K125 kg/ha;P26.2kg /ha K125 kg/ha;P52.3 kg /ha K125 kg/ha;P104.6 kg /ha	FALSE	—	40 kg N ha−1 and 20 kg Mg ha−1	Ginsengsaponin Ginsengsaponin Rg1 Ginsengsaponin Rb1 Ginsengsaponin Rb2 Ginsengsaponin Rc Ginsengsaponin Rd Ginsengsaponin Re

**Table 1B:** Characterization of the effect of N、P、K fertilizer addition on the saponin content of different medicinal plants.

	Authour	Test Object	Test type	Type of climate in the test site	Test location	Type of organic fertilizer added to the experiment	Type of inorganic fertilizer used in the experiment	Organic Fertilizer Base Fertilizer	Inorganic base fertilizer	Whether to apply additional fertilizer	Additional application of organic fertilizer	Additional application of inorganic fertilizers	Saponin types
14	Yang et al. (2022)	Ginseng	Potting experiment	Temperate continental monsoon climate	Wendeng District, Shandong Province, China	NPK:Biochar	—	0.6%(12g)1.2%(24g) 1.8%(32g) 2.4%(48g)	—	FALSE	—	—	Ginsengsaponin Rg1 Ginsengsaponin Rb1 Ginsengsaponin Rb2 Ginsengsaponin Rd Ginsengsaponin Re Ginsengsaponin Rh1 Ginsengsaponin Rh2
15	Ng et al. (2022)	Pseudostellariae Radix	Potting experiment	Subtropical highland monsoon humid climate	Bijie, Guizhou Province, China	NPK:Biochar Phosphorus modified biochar	—	3%Biochar(By mass) 5%Biochar(By mass) 3%Phosphorus modifiedBiochar(By mass) 5%Phosphorus modifiedBiochar(By mass)	—	—	FALSE	—	Pseudostellaria saponins
16	Ng et al. (2022)	Pseudostellariae Radix	Potting experiment	Subtropical highland monsoon humid climate	Bijie, Guizhou Province, China	NPK:Peanut shell biochar	—	3%Biochar(By mass) 5%Biochar(By mass)	—	FALSE	—	—	Pseudostellaria saponins
17	Yin et al. (2013)	Pseudostellariae Radix	Tissue culture tests	Subtropical highland monsoon humid climate	Guizhou Province, China	—	NK:NH_4_CL/KNO_3_	—	NH4CL:KNO3=0:60 NH4CL:KNO3=20:40 NH4CL:KNO3=30:30 NH4CL:KNO3=40:20 NH4CL:KNO3=60:0	FALSE	—	—	Ginsengsaponin
18	Cao et al. (2020)	American Ginseng	Field experiment	Temperate continental monsoon climate	Fusong County, Baishan, Jilin Province, China	—	N:UREA P:Calcium triple superphosphate K:potassium sulfate	—	N:50 kg/haP:460kg/haK:250 kg/ha N:10 g/m2P:460kg/haK:250 kg/ha N:20g/m2P:460kg/haK:250 kg/ha N:40g/m2P:460kg/haK:250 kg/ha	FALSE	—	—	Ginsengsaponin Rd Ginsengsaponin Re Panax quinquefolium saponins Rb3
19	Moon et al. (2018)	Codonopsis	Field experiment	Subtropical climate	Gyeongsang National University, Korea	NPK:Fermented cakes, mixed organic matter, and other chemical organic fertilizers	—	11kg/ha 100kg/ha 4.3kg/ha 4.2kg/ha 80kg/ha	—	TRUE	8kg/ha 150kg/ha 17kg/ha 1kg/ha 120kg/ha	—	Lancemaside A Lancemaside B Lancemaside D
20	Shams et al. (2014)	Dioscorea	Field experiment	Semi-arid climate	Research Farm, Faculty of Agriculture, Zanjan University, Iran	—	N:UREA Cu:copper sulfate	N:50kg/ha 100kg/ha 150kg/ha 200kg/ha Cu:10kg/ha 20kg/ha 30kg/ha	—	FALSE	—	—	Diosgenin
21	Li et al. (2023a)	Bupleurum chinensis DC.	Potting experiment	Temperate monsoon climate	Lin Agricultural University, Changchun, Jilin Province	—	N:UREA P:calcium superphosphate (Ca(OH)_2_) K:potassium sulfate	—	N:0.1377g/kgP:0.1089g/kgK:0.1644g/kg N:0.2754g/kgP:0.1089g/kgK:0.1644g/kg	TRUE	—	N:0.1377g/kgP:0.1089g/kgK:0.1644g/kg N:0.2754g/kgP:0.1089g/kgK:0.1644g/kg	Saikosaponin a Saikosaponin c Saikosaponin d
22	Sun et al. (2022a)	Bupleurum chinensis DC.	Bionic culture	Temperate monsoon climate	Lindian, Daqing, Heilongjiang Province, China	—	N:Ca(H2PO4)2·H2O	—	P:10kg/ha 20kg/ha	FALSE	—	—	Saikosaponin a Saikosaponin d
23	Duan et al. (2016)	Platycodon grandiflorus	Potting experiment	Subtropical Monsoon	Nanjing, Jiangsu Province, China	—	N:Ammonium nitrate P:Sodium phosphate monobasic S:potassium sulfate Hoagland's Nutrient Solution	—	N1(7.5) P1(0.25) K1(1.5) N2(15)P2(1)K2(3) N3(30) P3(2)K3(9)mmo/L	TRUE	—	—	Platycodon saponin d
24	Zhu and Zhang (2017)	Platycodon grandiflorus	Field experiment	Continental monsoonal climate	Boshan, Shandong Province, P.R. China	—	N:controlled release fertilizerCU controlled release fertilizerCRU CU+CRU	—	CU:113.75kg/ha CRU:104kg/ha CU+CRU:87.75kg/ha	FALSE	—	CU:61.25kg/ha CRU:56kg/ha CU+CRU:47.25kg/ha	Platycodon saponin
25	Li et al. (2023f)	RhizomaParidisChinensis	Potting experiment	Humid subtropical monsoon climate	Chongqing, Sichuan Province, China	—	N:Ammonium nitrate P:diammonium phosphate (chemistry) K:potassium sulfate dicalcium chloride	—	N:(NH4NO3) 0.15 g/kg、P: [( NH4)2HPO4] 0.12 g/kg KCl: K2SO4 100:0、75:25、50:50、25:75、0:100	FALSE	—	—	Polyphyllin I Polyphyllin II Polyphyllin III Polyphyllin IV
26	El-Serafy et al. (2021)	Quinoa	Field experiment	Tropical desert climate	Faculty of Agriculture, Al-Azhar University, Cairo	NPK: Green manure(Azolla+Moringa)	—	20%AE0.2L 20%MLE0.2L 20%AE+MLE0.2L	—	TRUE	0.2 kg(190 kg ha-1)	—	Quinoa saponin
27	Gui et al. 2024	Ginseng	Potting experiment	Temperate continental monsoon climate	Northeast Forestry University	NPK:Biogas residue (corn stalk and cow dung)	—	Fresh biogas residue30% Semi-decomposed biogas residue30% Mature biogas residue compost(30%、70%、100% (By mass)	—	FALSE	—	—	Ginsengsaponin
28	Wang et al. (2023)	Panax notoginseng	Field experiment	Subtropical Monsoon	Daliushu Village, Luxi County, Honghe Prefecture, Yunnan Province, China	—	Massive element water soluble fertilizer	—	48 kg/ha 、72 kg/ha 、96 kg/ha、120 kg/ha	FALSE	—	—	Ginsengsaponin Rd Ginsengsaponin Rb1 Ginsengsaponin Re Ginsengsaponin Rg1 Notoginsenoside R1
29	Su et al. (2024)	RhizomaParidisChinensis	Field experiment	Humid subtropical seasonal -wind climate	Nanping, Fujian Province, China	—	N:UREA P:calcium superphosphate (Ca(OH)_2_) K:potassium sulfate	—	N:135kg/ha 270kg/ha 105kg/ha P:187.5kg/ha 375kg/ha 562.5kg/ha K:150kg/ha 300kg/ha 450kg/ha	TRUE	—	Ratio of base fertilizer to topdressing fertilizer: N (5: 5), P (8: 2), K (5: 2: 3)	Polyphyllin I Polyphyllin II Polyphyllin III Polyphyllin IV

The table comprises 29 references on 10 medicinal plants containing saponins, three methods of fertilisation, and details of the location and type of climate, as well as the experimental treatments.

### 2.3 Quality assessment of research literature

**Figure 2:**
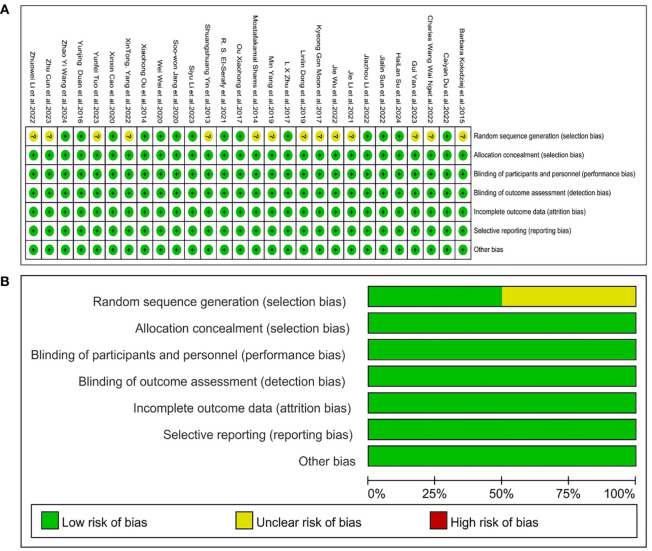
Risk of bias map for included studies. The risk of bias for the included studies was assessed using Review Manager 5.4. **(A)** Illustrates the specific risk assessment for each article with respect to each assessment aspect. **(B)** Illustrates the proportion of each risk for each assessment aspect of the included articles.

This meta-analysis employs Cochran’s Review Manager 5.4 software to evaluate the quality and risk of the literature. The assessment was conducted across six domains: selection bias, allocation bias, performance bias, measurement bias, follow-up bias, reporting bias, and other potential biases. Each criterion was evaluated and classified as either “low risk”, “unclear risk”, or “high risk” of bias (**Figure 2**).

### 2.4 Data analysis

First, the gathered data will be categorized and grouped based on the type of fertilizer applied and the type of medicinal plant saponin measured, with four distinct categories established: 1) the effect of fertilizer addition on medicinal plant saponin content; 2) the influence of inorganic fertilizer treatment on medicinal plant saponin content; 3) the effect of organic fertilizer treatment on medicinal plant saponin content; and 4) the impact of combined organic and inorganic fertilizer application on medicinal plant saponin content. Subsequently, the Meta-analysis data package of Stata MP 17 will be utilized for data analysis and the construction of forest plots. Given that the outcome measurement indicators in the included studies consist of continuous variables with varying scoring methods, the standardized mean difference (SMD) and corresponding 95% confidence interval from different studies will be employed as effect sizes for result aggregation. Additionally, the I^2^ statistic will be used to assess the heterogeneity of effects among the studies.

## 3 Results

### 3.1 Effect of fertilizer addition on the saponin content of medicinal plants

**Figure 3:**
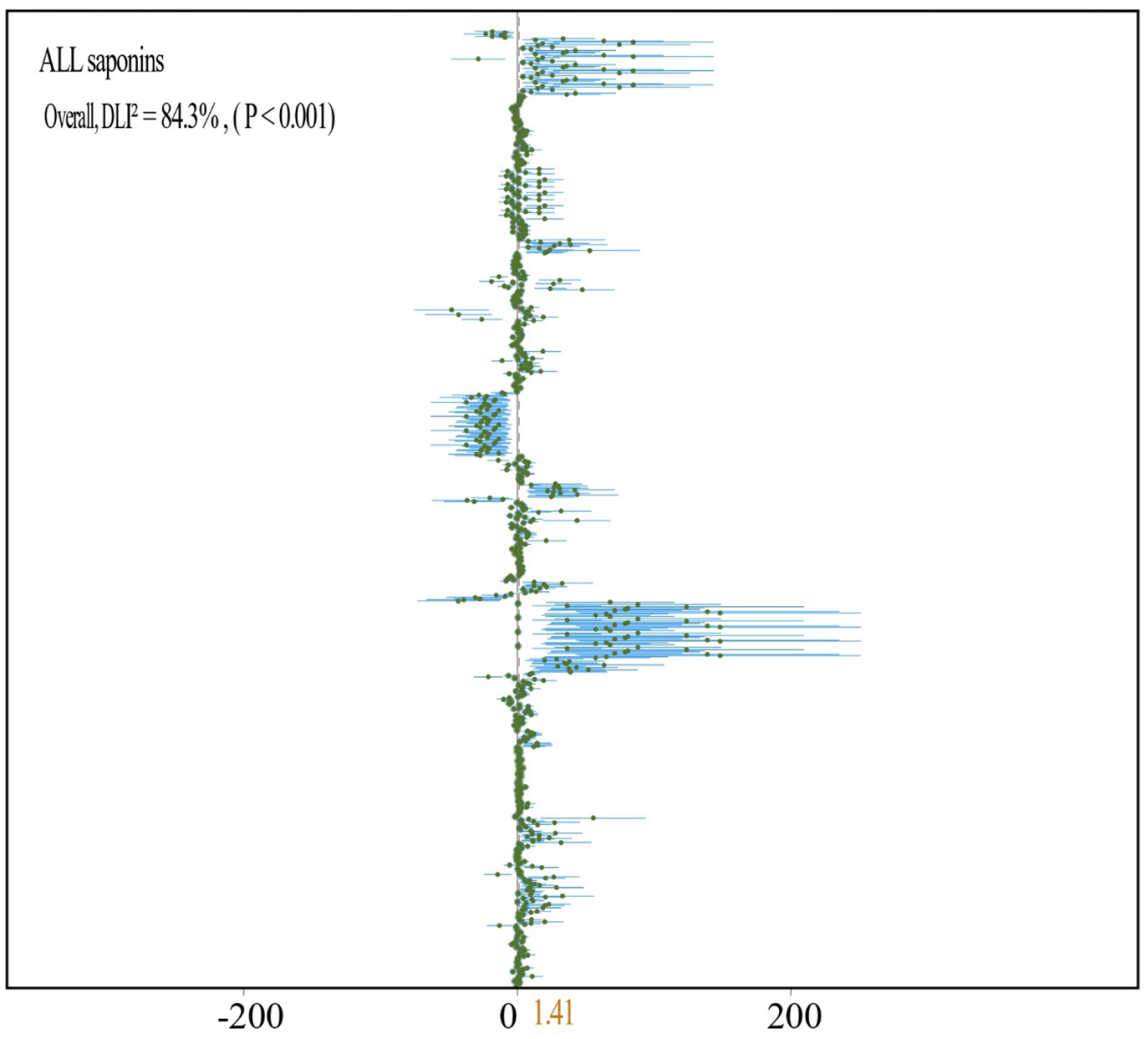
The overall forest plot showing the impact of fertilizer addition on the saponin content of medicinal plants. The plot is generated using a random-effects model, where N represents the number of studies included, and the numbers in parentheses indicate the total sample size.

**Figure 4:**
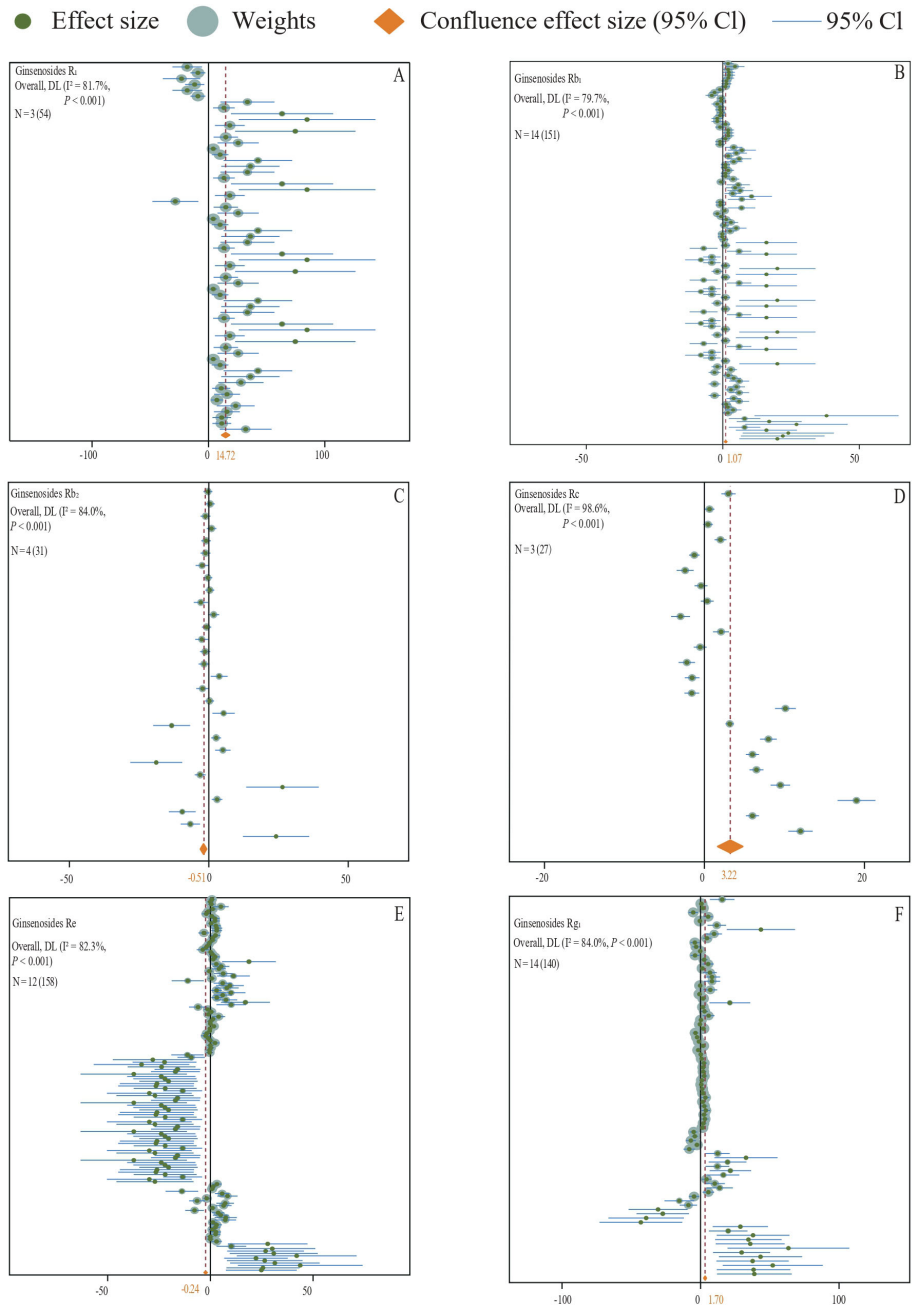
**(A–F)** A forest plot of the effect of all types of fertilizer additions on the content of medicinal plant saponins is presented. The plotting was conducted using a random effects model, where N indicates the number of included studies, and the number in parentheses indicates the total sample size.

**Figure 5:**
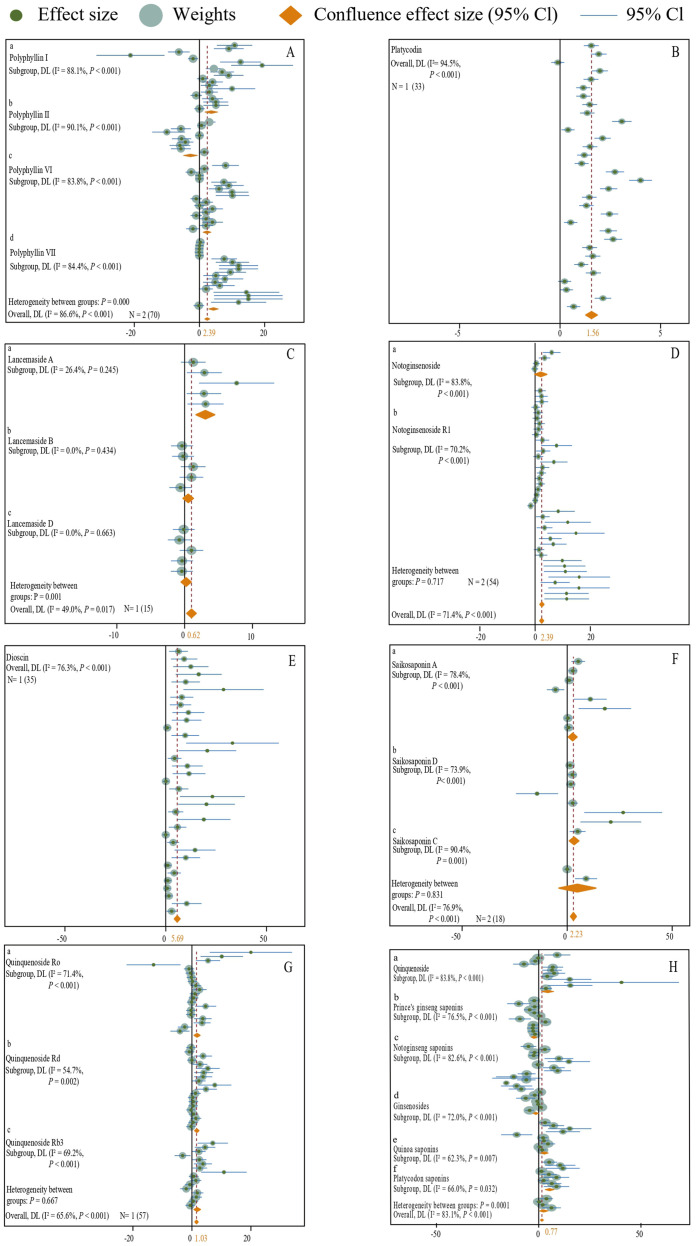
**(A–H)** A forest plot of the effect of all types of fertilizer additions on the content of medicinal plant saponins is presented. A random effects model was employed for plotting, with N indicating the number of included studies and the number in parentheses indicating the total sample size.

By analyzing the experimental data from all fertilizer groups, it has been demonstrated that the application of fertilizer markedly increases the saponin content in medicinal plants (N = 29,912; SMD = 1.41; *P* < 0.001) (**Figure 3**). Specifically, fertilizer application notably enhances the levels of Ginsenoside R1 (N = 3,54; SMD = 14.72, *P* < 0.001) (**Figure 4A**), Ginsenoside Rb1 (N = 14,151; SMD = 1.07, *P* < 0.001) (**Figure 4B**), Ginsenoside Rc (N = 3,27; SMD = 3.22, *P* < 0.001) (**Figure 4D**), Ginsenoside Rg1 (N = 14,140; SMD = 3.22, *P* < 0.001) (**Figure 4F**), Polyphyllin I, II, VI, VII (N = 2,70; SMD = 2.39, *P* < 0.001) (**Figure 5A**), Platycodin (N = 1,33; SMD = 1.56, *P* < 0.001) (**Figure 5B**), Lancemaside A, B, D (N = 1,15; SMD = 0.62, *P* < 0.05) (**Figure 5C**), Notoginsenoside and Notoginsenoside R1 (N = 2,54; SMD = 2.39, *P* < 0.001) (**Figure 5D**), Dioscin (N = 1,35; SMD = 5.69, *P* < 0.001) (**Figure 5E**), Saikosaponin A, C, D (N = 2,18; SMD = 2.23, *P* < 0.001) (**Figure 5F**), and Quinquenoside Ro, Rd, Rb3 (N = 1,57; SMD = 1.03, *P* < 0.001) (**Figure 5G**). Fertilizer application also markedly promotes the content of total saponins in medicinal plants, including *Ginseng* total saponins, *Panax notoginseng* total saponins, *Chenopodium quinoa Willd* total saponins, and *Pseudostellariae heterophylla* total saponins (N = 10,69; SMD = 0.79, *P* < 0.001) (**Figure 5H**). Moreover, the application of fertilizer markedly inhibits the accumulation of Ginsenoside Rb2 (N = 4,31; SMD = -0.51, *P* < 0.001) (**Figure 4C**), Ginsenoside Re (N = 12,158; SMD = -0.24, *P* < 0.001) (**Figure 4E**), and total saponins of *Codonopsis pilosula* (N = 2,12; SMD = -0.64, *P* < 0.001) (**Figure 5H**) in medicinal plants. The experimental data for *Lancemaside* and *Coix* saponins were derived solely from organic fertilizer treatments, whereas the data for *Polyphyllin, Dioscin, Quinquenoside*, and *Platycodin* originated from inorganic fertilizer treatments.

### 3.2 Effect of inorganic fertilizer addition on the saponin content of medicinal plants

**Figure 6:**
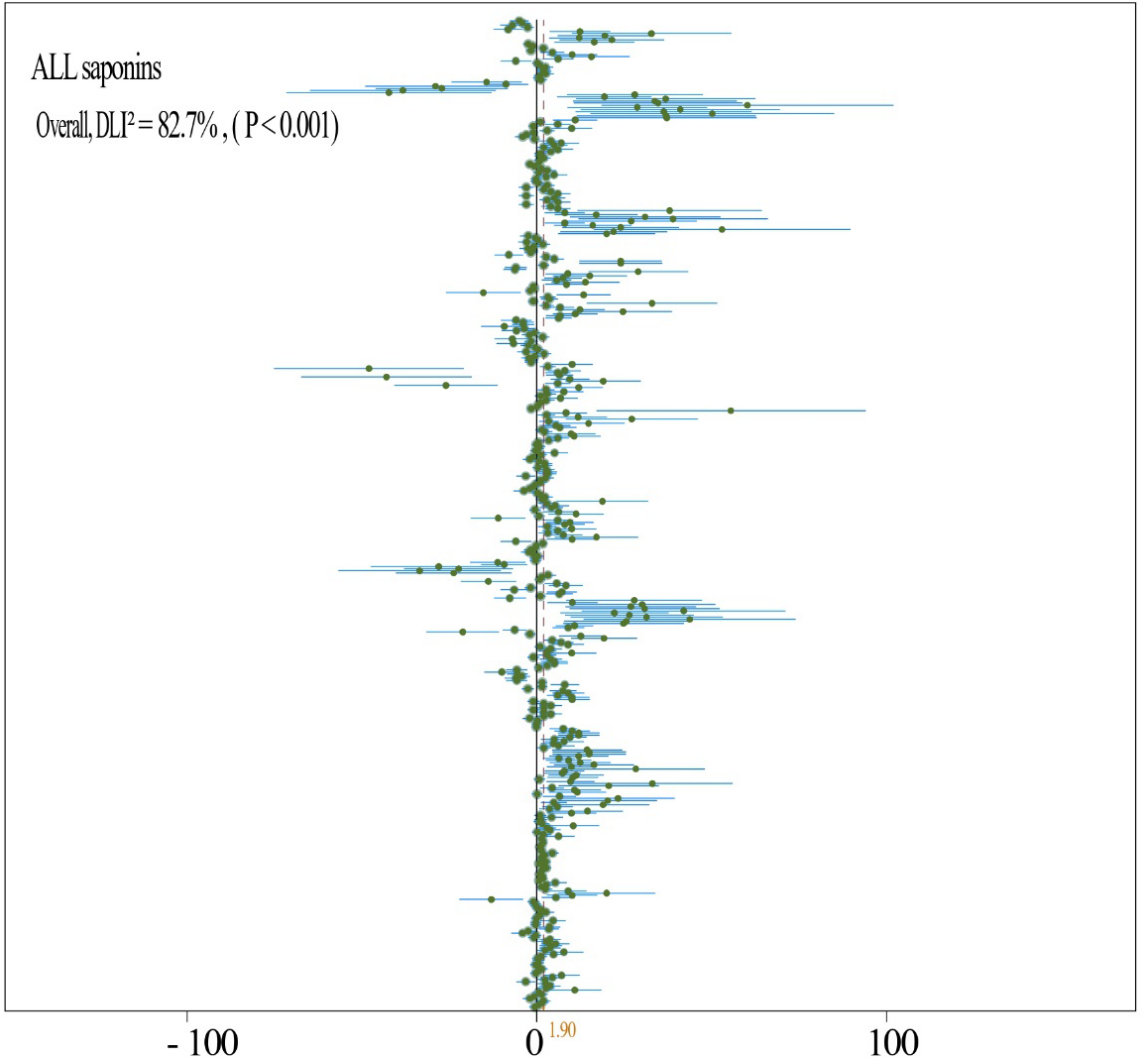
Total forest plot of the effect of different types of inorganic fertilizer addition on saponin content of medicinal plants saponins. A random effects model was used for plotting, where N indicates the number of included studies, and the number in parentheses indicates the total sample size.

**Figure 7:**
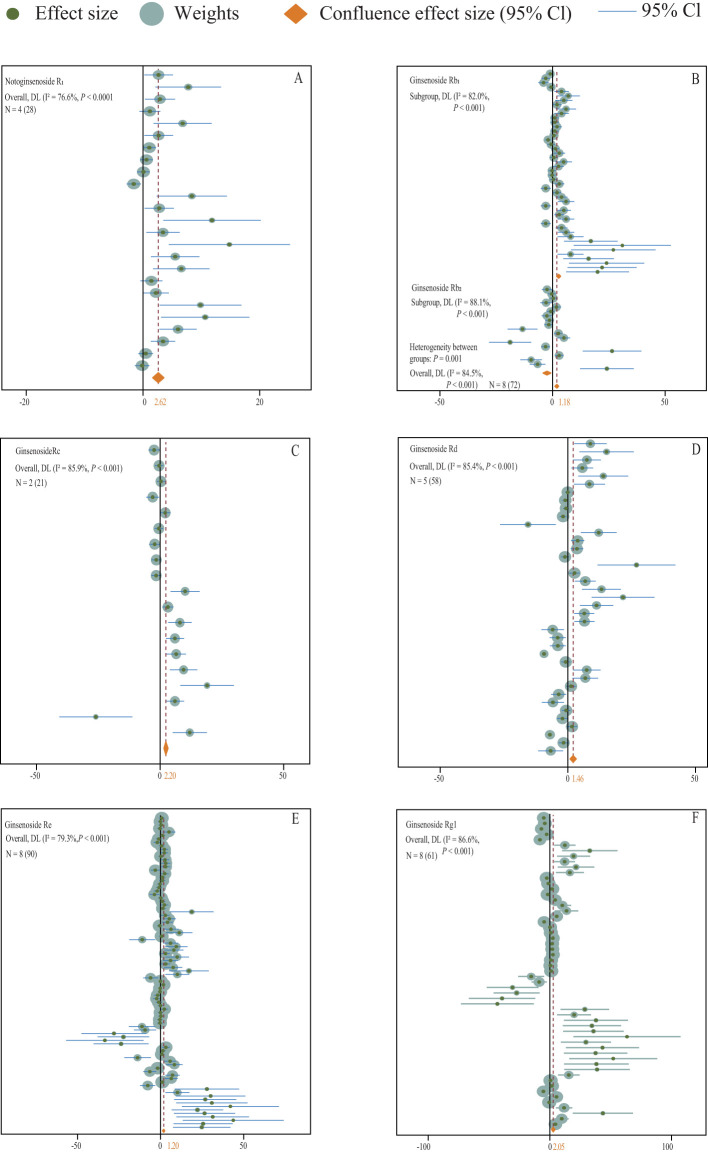
**(A–F)** A forest plot of the effect of inorganic fertilizer addition on the content of medicinal plant saponins is presented. A random effects model was employed for plotting, with N indicating the number of included studies and the number in parentheses indicating the total sample size.

The analysis of experimental data encompassing various types of inorganic fertilizers reveals a substantial increase in the accumulation of saponins in medicinal plants (N = 17,330; SMD = 1.90, *P* = 0.001) (**Figure 6**). Specifically, the application of inorganic fertilizers has been shown to markedly enhance the levels of Polyphyllin I, II, VI, VII (N = 2,70; SMD = 2.39, *P* < 0.001) (**Figure 5A**), Platycodin (N = 1,33; SMD = 1.56, *P* < 0.001) (**Figure 5B**), Dioscin (N = 1,35; SMD = 5.69, *P* < 0.001) (**Figure 5E**), Quinquenoside Ro, Rd, Rb3 (N = 1,57; SMD = 1.03, *P* < 0.001) (**Figure 5G**), Notoginsenoside R1 (N = 4,28; SMD = 2.62, *P* < 0.001) (**Figure 7A**), Ginsenoside Rb (N = 8,72; SMD = 1.18, *P* < 0.001) (**Figure 7B**), Ginsenoside Rc (N = 2,21; SMD = 2.20, *P* < 0.001) (**Figure 7C**), Ginsenoside Rd (N = 5,58; SMD = 1.46, *P* < 0.001) (**Figure 7D**), Ginsenoside Re (N = 8,90; SMD = 1.56, *P* < 0.001) (**Figure 7E**), and Ginsenoside Rg1 (N = 8,61; SMD = 2.05, *P* < 0.001) (**Figure 7F**) within medicinal plants.

### 3.3 Effect of organic fertilizer addition on the saponin content of medicinal plants

**Figure 8:**
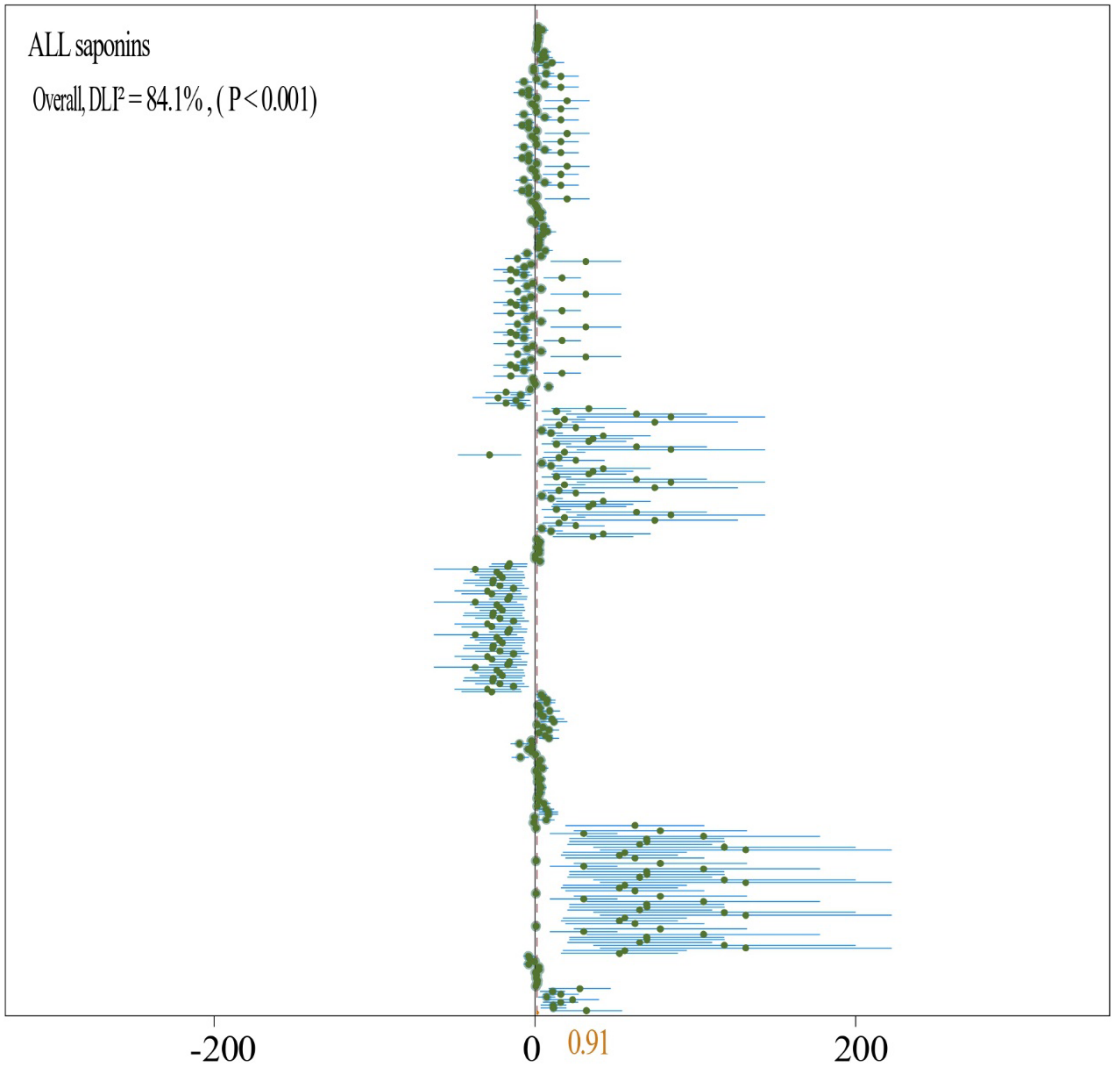
Total forest plot of the effect of different types of organic fertilizer addition on the saponin content of various medicinal plants. A random effects model was used for plotting, where N indicates the number of included studies, and the number in parentheses indicates the total sample size.

**Figure 9:**
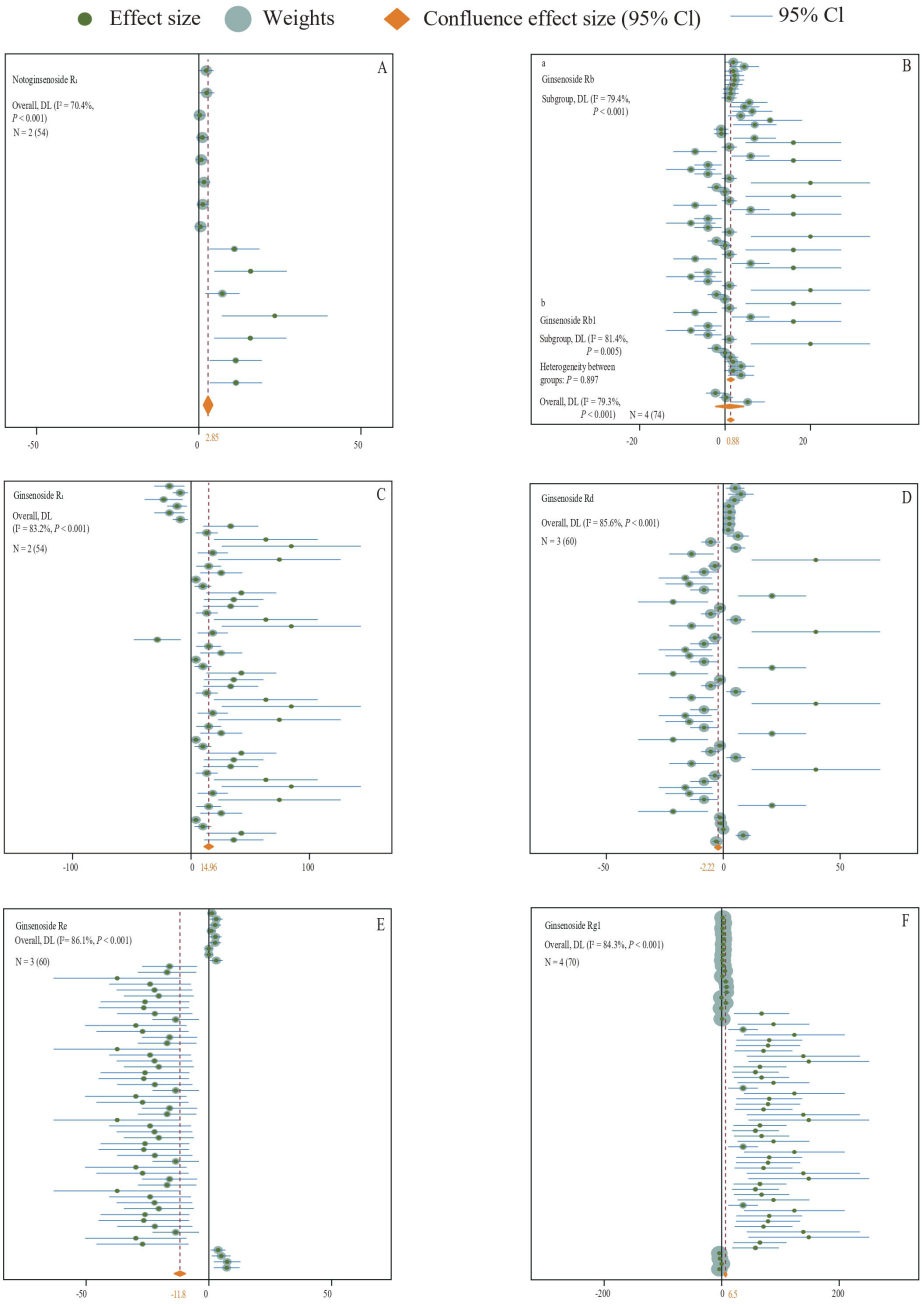
**(A–F)** The forest plot illustrates the effect of organic fertilizer addition on the content of medicinal plant saponins. A random effects model was employed for plotting, with N in the plot indicating the number of included studies and numbers in parentheses indicating the total sample size.

The analysis of experimental results involving the incorporation of organic fertilizers indicates that such an inclusion leads to a marked increase in saponin accumulation in medicinal plants (N = 11,372; SMD = 0.91, *P* < 0.001) (**Figure 8**). Specifically, the application of organic fertilizers has been shown to markedly enhance the accumulation of Lancemaside A, B, D (N = 1,15; SMD = 0.62, *P* < 0.05) (**Figure 5C**), Ginsenoside R1 (N = 2,54; SMD = 2.85, *P* < 0.001) (**Figure 9A**), Ginsenoside Rb (N = 4,74; SMD = 0.88, *P* < 0.001) (**Figure 9B**), Ginsenoside R1 (N = 2,54; SMD = 14.96, *P* < 0.001) (**Figure 9C**), and Ginsenoside Rd (N = 4,70; SMD = 6.5, *P* < 0.001) (**Figure 9F**), while suppressing the accumulation of Ginsenoside Rd (N = 3,60; SMD = -2.22, *P* < 0.001) (**Figure 9D**) and Ginsenoside Re (N = 3,60; SMD = -11.8, *P* < 0.001) (**Figure 9E**).

### 3.4 The effect of organic and inorganic fertilizers on the saponin content of medicinal plants

**Figure 10:**
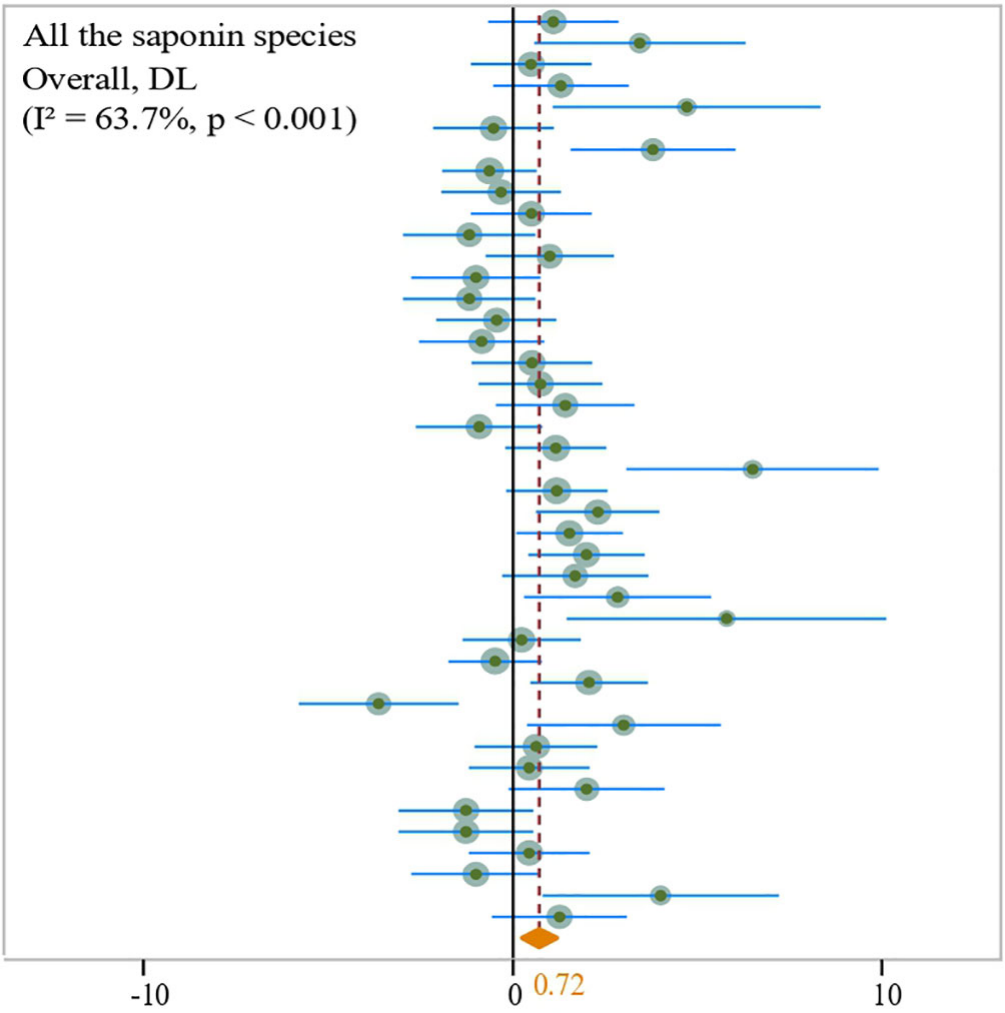
Total forest plot of the effect of organic and inorganic fertilizer formulation on saponin content of medicinal plants, plotted using random effects model.

**Figure 11:**
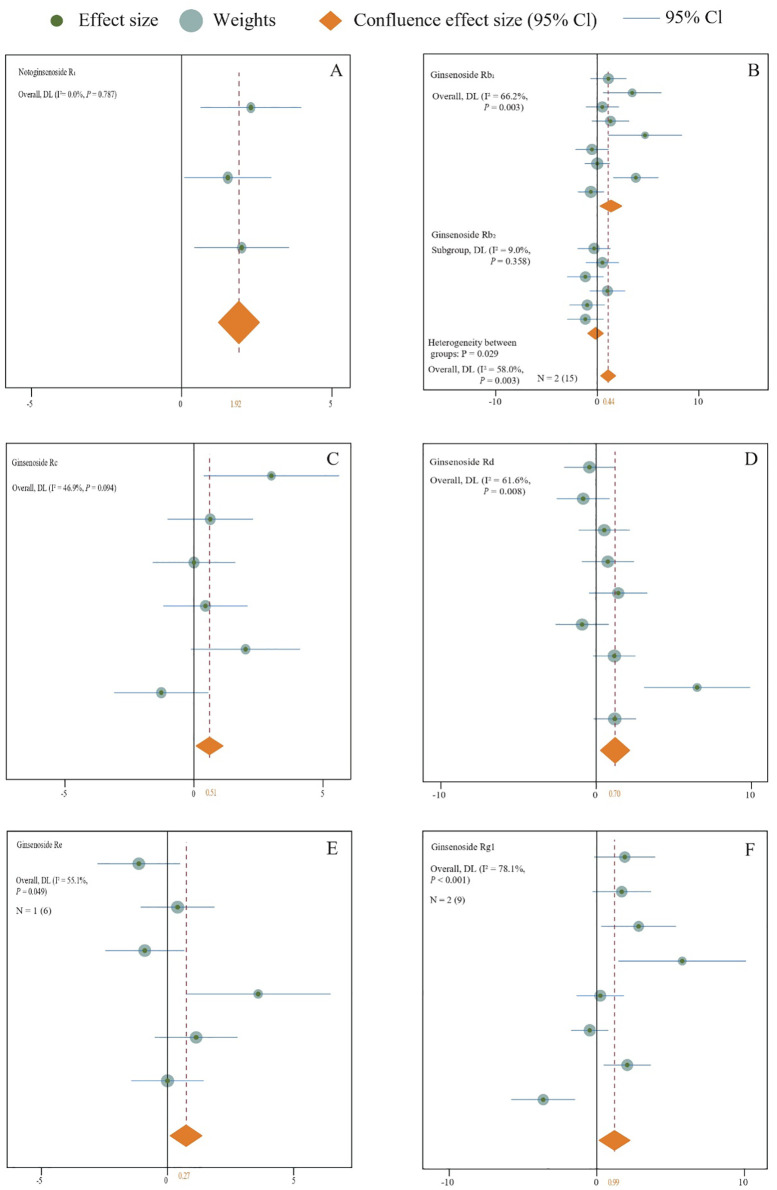
**(A–F)** The following forest plot illustrates the effect of organic and inorganic fertilizer formulations on the content of medicinal plant saponins. A random-effects model was employed for plotting, with N in the figure indicating the number of included studies and numbers in parentheses indicating the total sample size.

The assessment of experimental interventions that involve the concurrent use of both organic and inorganic fertilizers indicates that their combined application can notably promote the accumulation of saponins in medicinal plants (N = 2,43; SMD = 0.72, *P* < 0.001) (**Figure 10**). Specifically, for Notoginsenoside Rb1 (N = 1,3; SMD = 1.92, *P* < 0.001) (**Figure 11A**), Ginsenoside Rb (N = 2,15; SMD = 0.44, *P* < 0.001) (**Figure 11B**), Ginsenoside Rc (N = 2,6; SMD = 0.51, *P* < 0.001) (**Figure 11C**), Ginsenoside Rd (N = 2,9; SMD = 0.70, *P* < 0.001) (**Figure 11D**), Ginsenoside Rc (N = 1,6; SMD = 0.27, *P* < 0.001) (**Figure 11E**), and Ginsenoside Rg1 (N = 2,9; SMD = 0.99, *P* < 0.001) (**Figure 11F**).

## 4 Discussion

### 4.1 Effect and mechanism of inorganic fertilizer addition on medicinal plant soaps

Inorganic fertilizers are extensively utilized as a primary fertilization approach to improve both crop yield and quality (Melaku et al., 2018; Huang et al., 2022). For example, Xia et al. (2016) demonstrated that the application of inorganic fertilizers containing nitrogen, phosphorus, and potassium at respective rates of 0 kg 667 m², 17.01 kg 667 m², and 56.87 kg 667 m² resulted in the highest recorded root yield of 1861.90 g, alongside a reduction in the incidence of root diseases. Additionally, Sun et al. (2022b) observed that a combination of nitrogen fertilizer at 50 g m², phosphorus fertilizer at 15 g m², and potassium fertilizer at 60 g m² yielded the most significant benefits, notably increasing both the biomass of ginseng roots and the concentrations of several Ginsenosides (Rg1, Re, Rf, Rg2, Rb1, Ro, Rc, Rb2, Rb3, and Rd). These findings align with our results, which suggest that the incorporation of inorganic fertilizers substantially enhances the accumulation of various saponins, including Notoginsenoside Rg1, Ginsenoside (Rb1, Rc, Rd, Re, Rg1), as well as Polyphyllin, Dioscin, ginseng saponins, and Platycodin, in medicinal plants. The mechanisms responsible for the increased saponin content in Chinese medicinal plants due to inorganic fertilizer application may involve the following factors:

1) Direct nutrient supply: Inorganic fertilizers are capable of rapidly and directly delivering essential nutrients to plants, such as nitrogen, phosphorus, and potassium, which serve a pivotal function in the growth, development, and metabolic processes of medicinal plants (Li et al., 2021b; Dlamini et al., 2024). Additionally, specific inorganic fertilizers are also enriched with trace elements, including zinc, iron, and manganese, which are vital for the growth and metabolic activities of these plants (Manzoor et al., 2024). The biosynthesis of saponins in medicinal plants is closely associated with the availability of these key nutrients (Lu et al., 2024; Qin et al., 2024).

2) Enhancement of plant metabolism: Inorganic fertilizers are known to influence plant growth and development by modulating the synthesis and regulation of plant hormones (Adhikari et al., 2024; Wang et al., 2024c). Nitrogen-based fertilizers, for instance, can elevate the levels of gibberellins and auxins in plants, both of which are involved in promoting the biosynthesis of saponins (Leilah and Khan, 2021; Chen et al., 2024b). Furthermore, inorganic fertilizers can regulate saponin synthesis through modulation of the enzymatic activity involved in plant metabolic pathways (Ibrahim et al., 2013; Mohamed et al., 2023). Phosphorus, an essential element in numerous enzyme synthesis processes, also affects the activity of these enzymes, thereby influencing saponin production (Gui et al., 2024; Shi et al., 2024; Solangi et al., 2024). In summary, the application of inorganic fertilizers provides critical nutrients for the growth of medicinal plants within a relatively brief period, facilitating high yields. Additionally, the incorporation of inorganic fertilizers can modulate the activity of soil enzymes and phytohormone synthases, thus enhancing the concentration of bioactive constituents in medicinal plants and improving their overall quality (Qaisi et al., 2023; Zhang et al., 2022). However, prolonged use of inorganic fertilizers may lead to detrimental effects, such as a decline in soil organic matter content, soil acidification, and a reduction in soil microbial diversity (Tripathi et al., 2020; Pahalvi et al., 2021), which can adversely affect the growth of medicinal plants, the synthesis of their active compounds, and subsequently, their yield and quality (Pant et al., 2021; Ouyang et al, 2024). This could compromise the sustainable cultivation of medicinal plants.

### 4.2 Effect and mechanism of organic fertilizer addition on medicinal plant saponins

Organic fertilization, a widely adopted agricultural practice to improve crop quality, is commonly applied in the cultivation of medicinal plants to enhance the concentration of bioactive constituents (Singh et al., 2020; Tian et al., 2022). In contrast to the rapid nutrient release characteristic of inorganic fertilizers, organic fertilizers gradually release essential nutrients into the soil, thereby ensuring a continuous supply for plant growth and the synthesis of secondary metabolites (Wang et al., 2021; Shaji et al., 2021). Furthermore, the application of organic fertilizers has been shown to increase the population of soil microorganisms and stimulate soil enzyme activity, thereby influencing nutrient transformations in the soil and facilitating the uptake of available nutrients by plants (Řezáčová et al., 2021; Shu et al., 2022; Hu et al., 2024). The results of this study reveal that the incorporation of organic fertilizers markedly enhances the content of Ginsenosides (Rg1, Rb1, Rb2, and Re) and Notoginsenoside Rg1. This finding aligns with those of Liu et al. (2024b), who reported that, compared to the control group, treatments with 2% and 4% organic fertilizer increased the content of polyphylla saponins by 37.1% and 60.3%, respectively. In a similar vein, Gui et al. (2024) observed that the application of fully matured, decomposed biogas slurry notably increased the total saponin content of ginseng. The proposed mechanisms underlying these promoting effects are as follows:

1) The application of organic fertilizers has been shown to enrich the population of beneficial soil microorganisms and to enhance the saponin content in medicinal plants by improving the soil environment (Cheng et al., 2020). For instance, Shi et al. (2022) demonstrated that the use of bio-organic fertilizers resulted in a significant increase in the dry weight of *Panax notoginseng* compared to the control treatment. This outcome was attributed to the ability of bio-organic fertilizers to mitigate soil acidification, increase organic matter content, and elevate the levels of both total and available nutrients in the soil. Moreover, bio-organic fertilizers had a profound impact on the composition of rhizosphere bacterial communities, promoting the proliferation of specific bacterial groups. In a similar study, Du et al. (2024) reported that the addition of chicken manure reduced the NH_4_^+^/NO_3_^-^ ratio in the soil by 64%, leading to increased activity of saprophytic fungi (Sordariales and Pezizales), which consequently enhanced the yield of *Fritillaria* bulbs by a factor of 6.8 and elevated the content of active substances. In essence, organic fertilizers establish an optimal nutrient environment for soil microorganisms, thereby enhancing their abundance and activity, including that of actinomycetes, nitrifying bacteria, phosphate-solubilizing bacteria, and other microorganisms (Hou et al., 2023). These microorganisms serve a critical function in facilitating nutrient conversion in the soil and supporting plant nutrient uptake, ultimately promoting the synthesis of saponins in medicinal plants. Furthermore, Li et al. (2023c) demonstrated that co-inoculation of *Bacillus amyloliquefaciens*, *Bacillus weihenstephanensis*, and *Paenibacillus mucilaginosus* in the cultivation of *Paris polyphylla* resulted in increases in root and rhizome biomass, as well as in the content of steroidal saponins, available phosphorus, and total phosphorus by 134.58%, 132.56%, 51.64%, and 17.19%, respectively.

2) Organic fertilizer application has been shown to enhance soil enzyme activity, thereby contributing to the accumulation of bioactive compounds in medicinal plants. Enzymes such as invertase, urease, and peroxidase are essential in the breakdown of organic matter and the transformation of nutrient elements within the soil, which in turn improves nutrient uptake by plants (Liu et al., 2024b). For instance, a study indicated that the use of a bio-organic fertilizer containing *Bacillus megaterium*, *Bacillus mucilaginosus*, and *Bacillus subtilis* resulted in a significant increase in the activity of various soil enzymes, including leucine aminopeptidase, β-glucosidase, β-N-acetylglucosaminidase, soil acid phosphatase, β-xylosidase, and β-mannosidase (*P* < 0.05). This treatment also led to a 17.71% increase in tea polyphenol content, as well as a 33.05% and 22.20% increase in amino acid concentrations (Liu et al., 2023b). In a separate study by Jiang et al. (2024), the addition of 20 mg g⁻¹ and 40 mg g⁻¹ of biochar resulted in elevated levels of available potassium, phosphorus, nitrogen, and organic matter, as well as increased urease and protein activity, which subsequently raised the total flavonoid content in *Hedyotis diffusa*.

Moreover, while the application of organic fertilizers can facilitate the accumulation of bioactive compounds in medicinal plants, consideration must be given to potential limitations (Shaji et al., 2021; Tian et al., 2022). Organic fertilizers, characterized by low nutrient concentrations, require microbial decomposition for nutrient absorption and utilization by plants, which can negatively affect plant growth and development (Hubballi et al., 2022; Hong et al., 2023; Tao et al., 2024). In addition, the long-term application of organic fertilizers may disrupt soil ecology. For example, such fertilizers may contain residual heavy metals, antibiotics, veterinary drugs, and other contaminants (Xue et al., 2021; Shu et al., 2024). Therefore, the adoption of scientifically sound and appropriate methods for organic fertilizer use is essential to support the sustainable cultivation of Chinese medicinal plants.

### 4.3 Effect of organic and inorganic fertilizer blending on saponins of medicinal plants

The combined use of organic and inorganic fertilizers, regarded as an optimized fertilization strategy, not only amalgamates the advantages of both fertilizer types but also mitigates the limitations inherent in their independent applications (Shang et al., 2025). This integrated approach addresses the decline in soil microbial populations caused by the prolonged use of inorganic fertilizers alone while also overcoming the slow nutrient release characteristic of organic fertilizers when applied independently (Wang et al., 2024a). Moreover, the ability to adjust substitution ratios offers varied options for soil improvement across different environmental contexts, providing flexible strategies for nutrient supply tailored to specific crop cultivation methods (Song et al., 2022; Xu et al., 2024). The practice of integrated fertilization has been widely adopted in the cultivation of medicinal plants and has been extensively studied (Nchu et al., 2017; Tripathi and Singh, 2021). The results of this investigation revealed that the concurrent use of organic and inorganic fertilizers markedly enhanced the concentrations of Ginsenosides Rb1, Rb2, Rd, and Re in medicinal plants. These findings align with those reported by Li et al. (2023b), who demonstrated that a 7:3 mixture of organic and inorganic fertilizers improved soil fertility and increased Gorgon fruit yield by 5.72–6.21% relative to the control group. Additionally, Li et al. (2023b) found that this 7:3 fertilizer ratio notably increased the abundance of beneficial microorganisms, such as *Chloroflexi*, *Gammaproteobacteria*, and *Hypocreales-incertae-sedis*. Likewise, Li et al. (2023d) indicated that replacing chemical fertilizers with organic fertilizers boosted crop yield in a wheat-maize rotation system, and partial substitution of chemical fertilizers with organic alternatives improved soil humus quality, reduced soil acidification, and enhanced soil enzyme activities, with the optimal substitution ratio being 50%. Furthermore, Sun et al. (2024) reported that the combined application of organic and inorganic fertilizers resulted in varying increases in the content of total sugars, starch, crude protein, total amino acids, and ash. The highest improvements were observed with a treatment consisting of 25% organic fertilizer and 75% inorganic fertilizer, showing increases of 6.31%, 3.78%, 18.40%, 29.70%, and 10%, respectively. Finally, Ou et al. (2017) identified that a 1:1 ratio of organic to inorganic nitrogen was optimal for promoting the growth of *Panax notoginseng* and the accumulation of saponins, with this ratio markedly increasing the total content of *Notoginsenoside.*

Finally, the combined application of organic and inorganic fertilizers has the potential to improve soil quality and promote plant growth through various mechanisms, which may influence the saponin content in medicinal plants (Du et al., 2023). This effect results from the interaction of multiple factors, including enhanced soil fertility, the optimization of soil microbial communities, the direct provision of essential nutrients for plant development, and the enhancement of the plant growth environment (Trivedi et al., 2020; Ahsan et al., 2024; Qiao et al., 2024)

## 5 Conclusions

This meta-analysis presents evidence that the incorporation of various fertilizers notably increases the accumulation of saponins in medicinal plants. Organic fertilizers have been shown to consistently elevate the levels of saponins, such as Notoginsenoside R1, Ginsenoside Rb1, Ginsenoside Rb2, Ginsenoside Re, Ginsenoside Rg1, Lancemasid saponins, and Quinoa saponins while reducing the concentration of Ginsenoside Rc and Ginsenoside Rd. The application of inorganic fertilizers has similarly been observed to substantially enhance the accumulation of a wide range of saponins, including Notoginsenoside Rg1, Ginsenoside Rb1, Ginsenoside Rc, Ginsenoside Rd, Ginsenoside Re, Ginsenoside Rg1, Polyphyllin, Dioscin, Quinquenoside, and Platycodin. Furthermore, the combined application of organic and inorganic fertilizers markedly boosts the levels of Notoginsenoside R1, Ginsenoside Rb1, Ginsenoside Rb2, Ginsenoside Rc, Ginsenoside Rd, Ginsenoside R, and Ginsenoside Rg1 in medicinal plants. Our research provided a scientific evidence and guidance for the optimal selection and application of fertilizers in the cultivation of saponin-containing medicinal plants. Additionally, this investigation primarily focused on examining the effects of various fertilizers on saponin content in medicinal plants without delving into the underlying mechanisms. Additionally, this investigation primarily concentrated on examining the macroscopic effects of various fertilizers on saponin content in medicinal plants without addressing microscopic mechanisms. Future research could further explore the specific molecular mechanisms through which the combined use of organic and inorganic fertilizers influences gene expression related to saponin biosynthesis, plant growth hormone regulation, and soil microbial ecology, employing techniques such as field trials, soil culture experiments, plant physiology, and molecular biology methods,
